# Supplementary material for: Oncogenic Signalling of PEAK2 Pseudokinase in Colon Cancer
Source: Cancers (Basel). 2022 Jun 16;14(12):2981. doi: 10.3390/cancers14122981 (PMC9221080; doi:10.3390/cancers14122981)
Supplement: Supplementary file 1 [file cancers-14-02981-s001.zip › cancers-1751392-supplementary.pdf]

cancers

MDPI

Supplementary Materials: Oncogenic Signalling of PEAK2 Pseudokinase in Colon Cancer

Céline Lecointre, Elise Fourgous, Ingrid Montarras, Clément Kerneur, Valérie Simon, Yvan Boublik, Débora Bonenfant, Bruno Robert, Pierre Martineau and Serge Roche

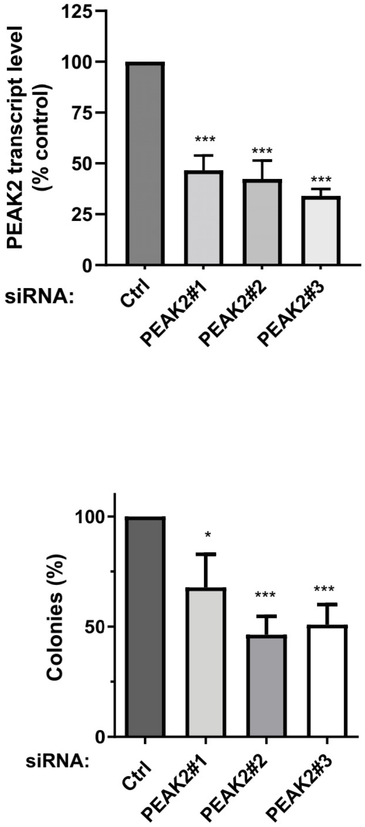

Figure S1. effect of siRNAs targeting PEAK2 expression colonies formation in.the Lovo CRC cells in soft agar. A relative PEAK2 transcript level in cells transfected with indicated siRNA. B. Anchorage-independent growth in soft agar of CRC cells transfected with indicated siRNA.). Is shown mean ± SEM; n = 3; *p < 0.05; **p < 0.01; ***p < 0.001 (Student's *t* test).

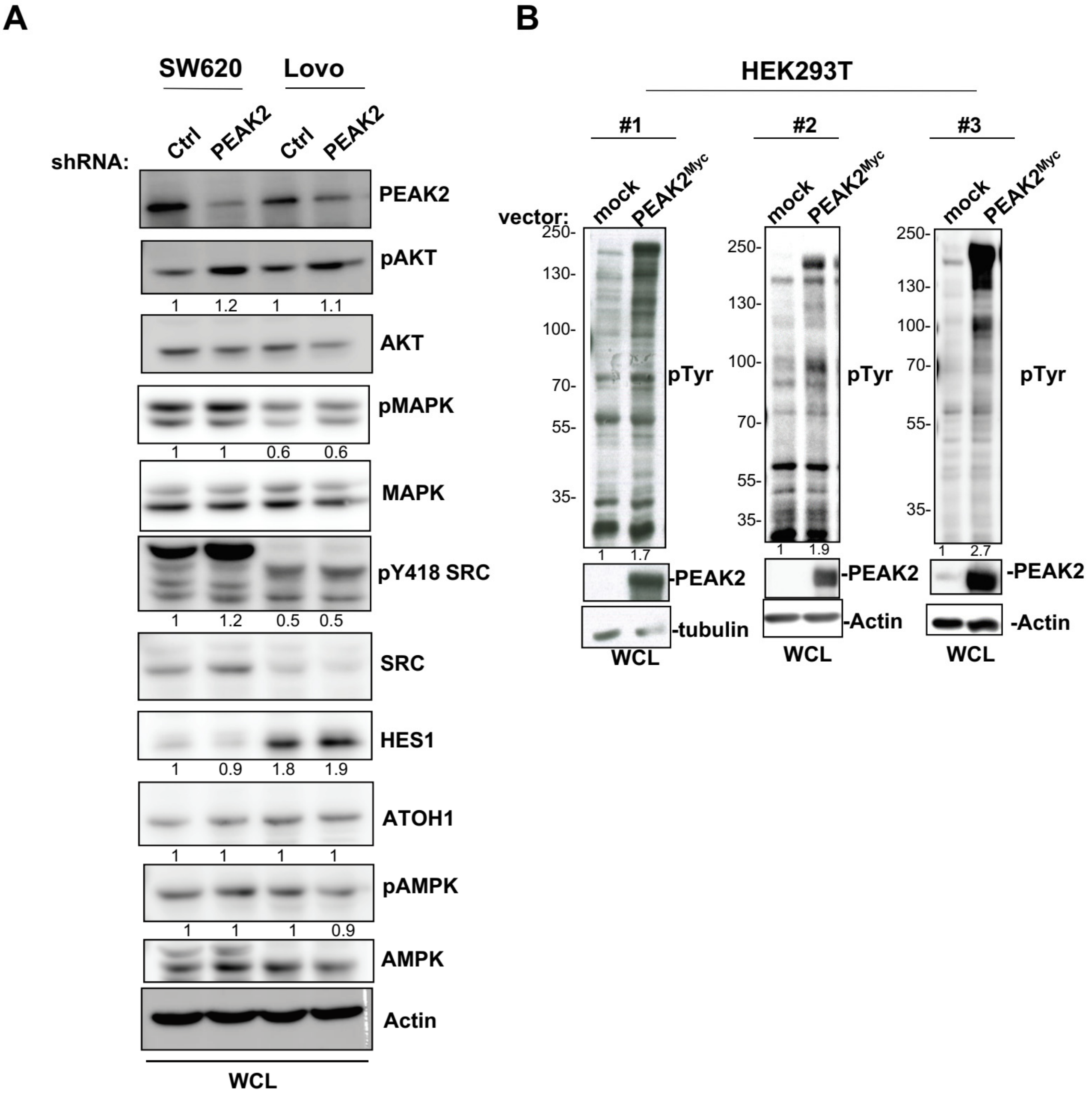

Figure S2. PEAK2 depletion does not affect RTK or Notch signaling in CRC cells. Is shown MAPK, AKT, Src, Notch and AMPK activities in CRC cells depleted or not PEAK2.

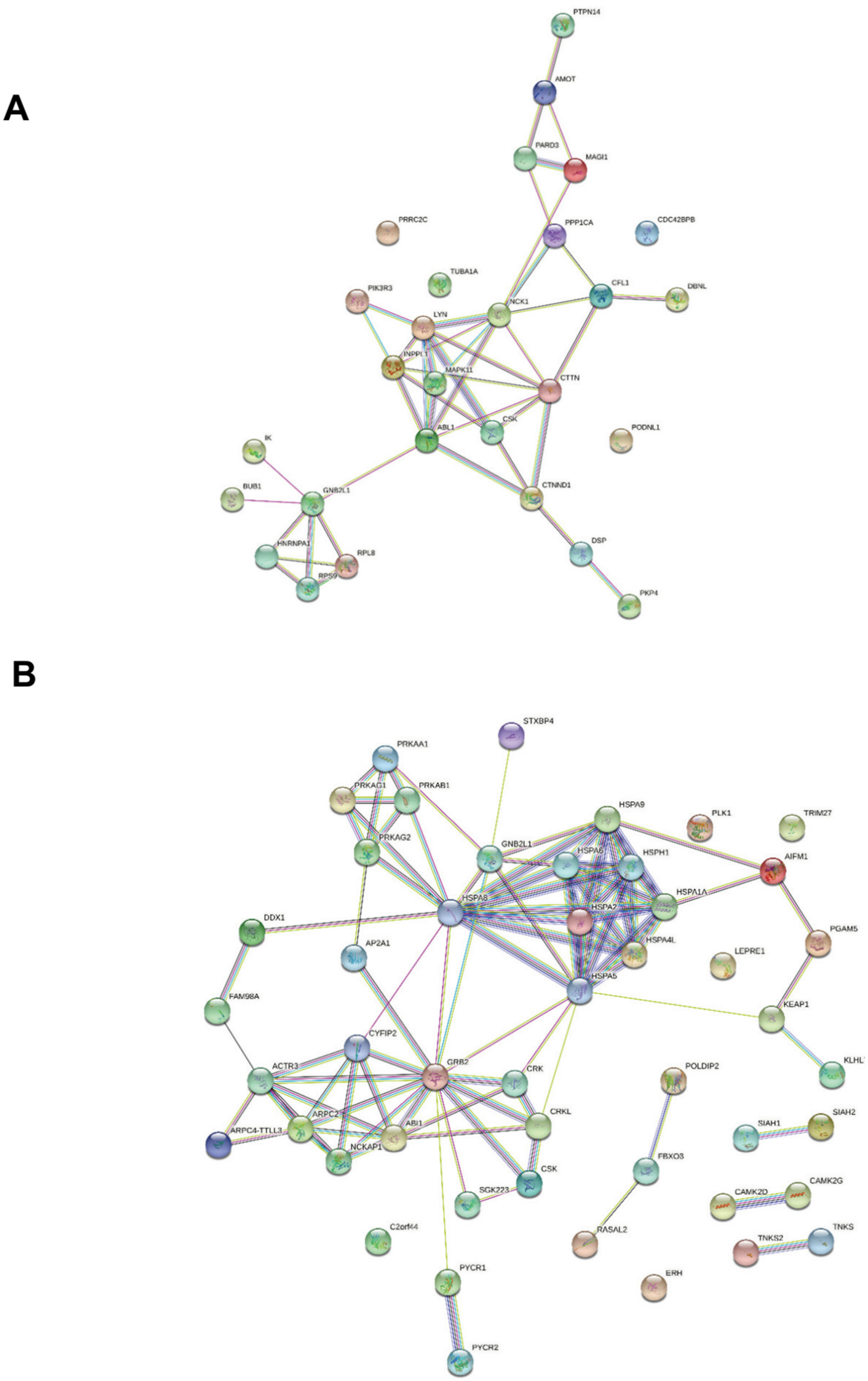

Figure S3. string analysis of PEAK2 phospho-proteomic (A) and interactomic analysis (50 main hits) (B). This analysis reveals regulators of F-actin assembly as important components of PEAK2 phospho-signaling.

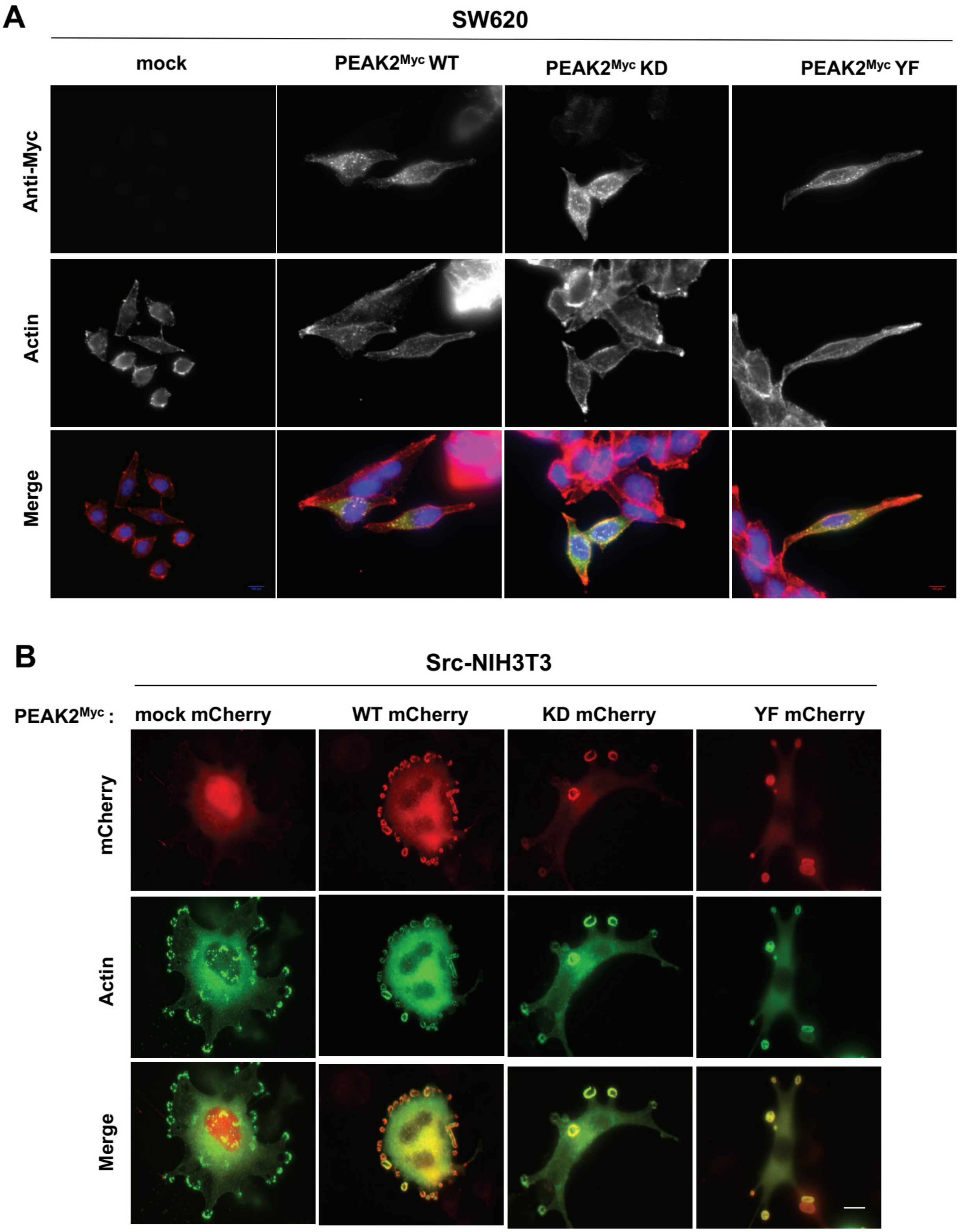

Figure S4. PEAK2 co-localizes with F-actin structures at focal adhesion of transformed cells. Immunostaining of PEAK2Myc and F-actin in SW620 andSrcYFNIH3T3.

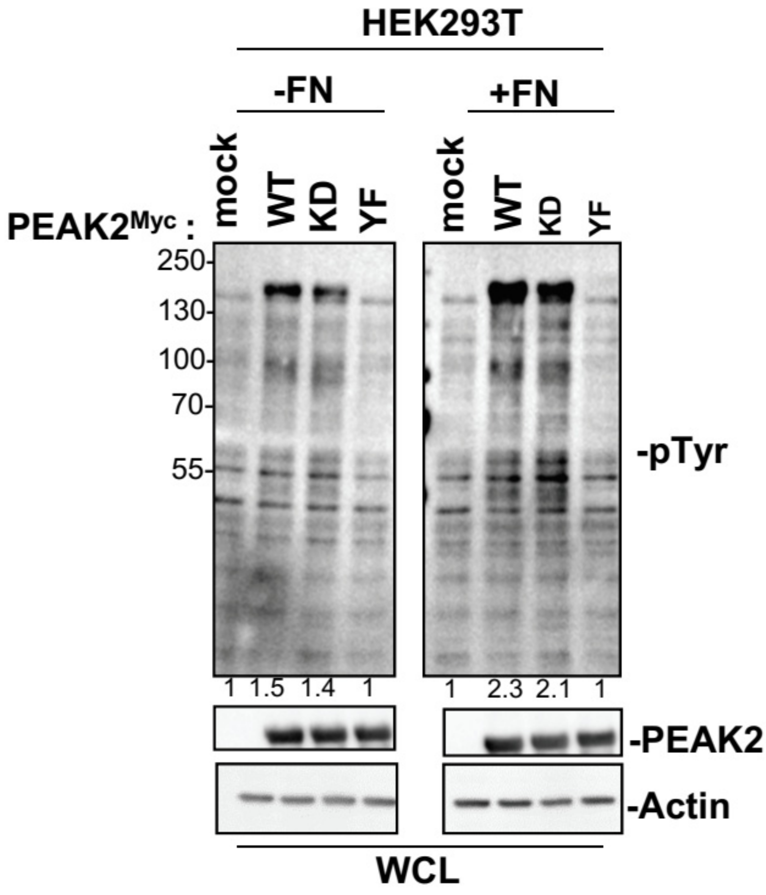

Figure S5. Fibronectin induces Y413-dependent PEAK2 phospho-tyrosine signaling. Protein tyrosine phosphorylation induced by indicated PEAK2 mutants transiently transfected in HEK293T cells that were coated or not with fibronectin (FN).

Figure 1A

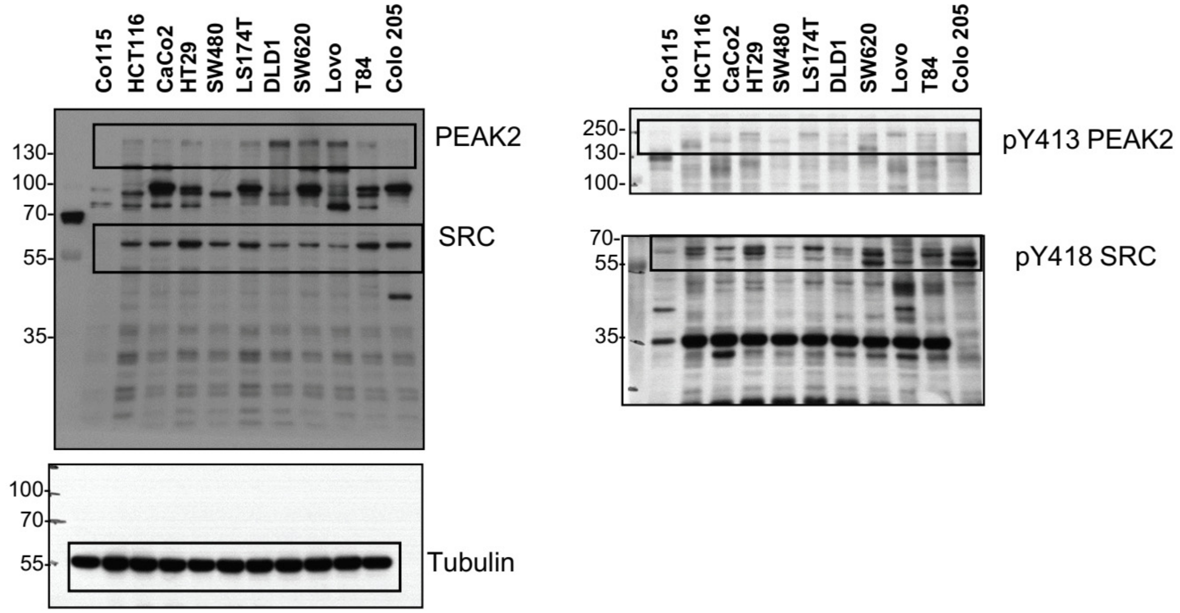

Figure 1B

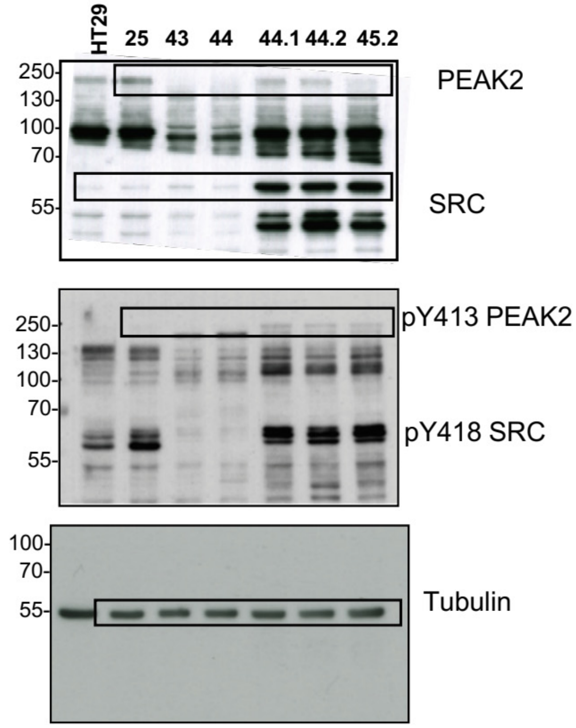

Figure 2

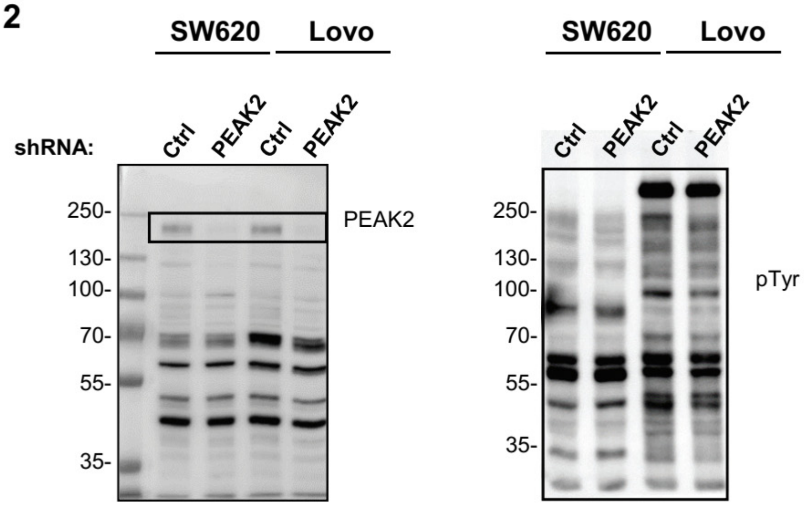

Figure 4A

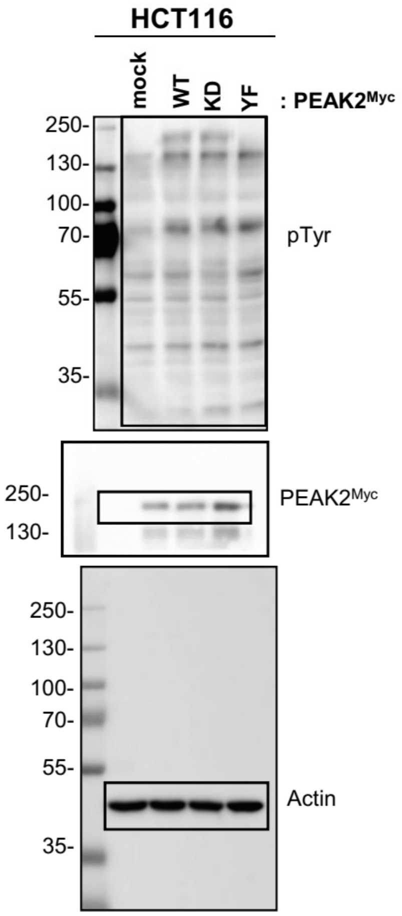

Figure 4C

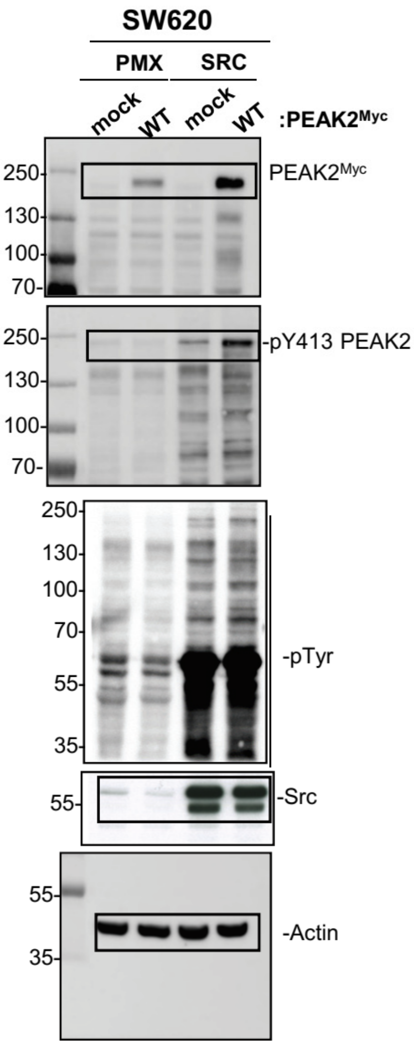

Figure 5C

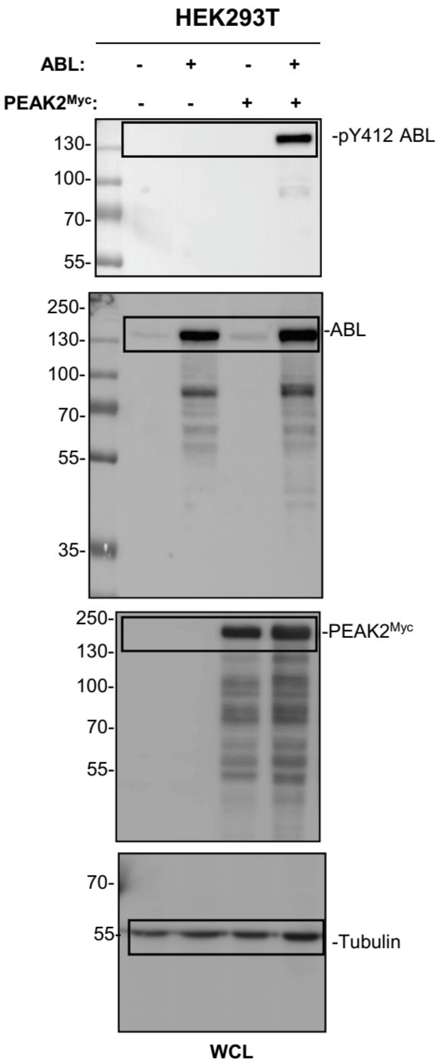

Figure 5D

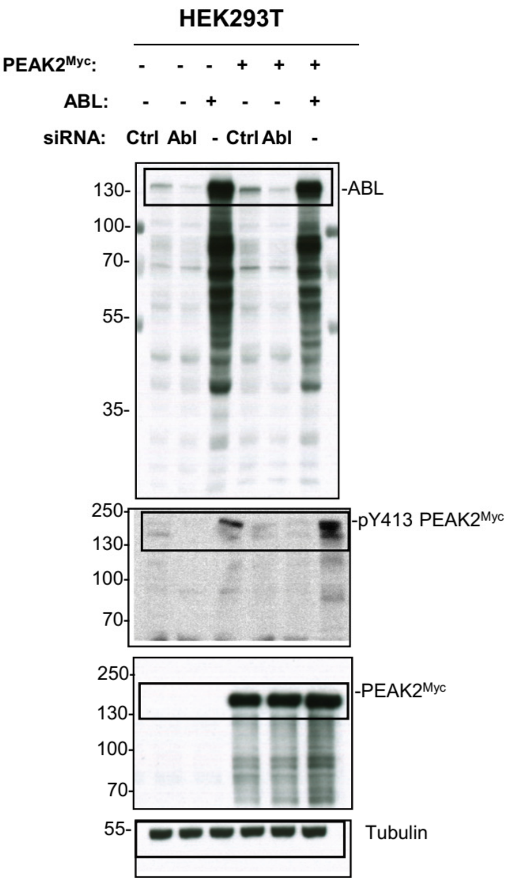

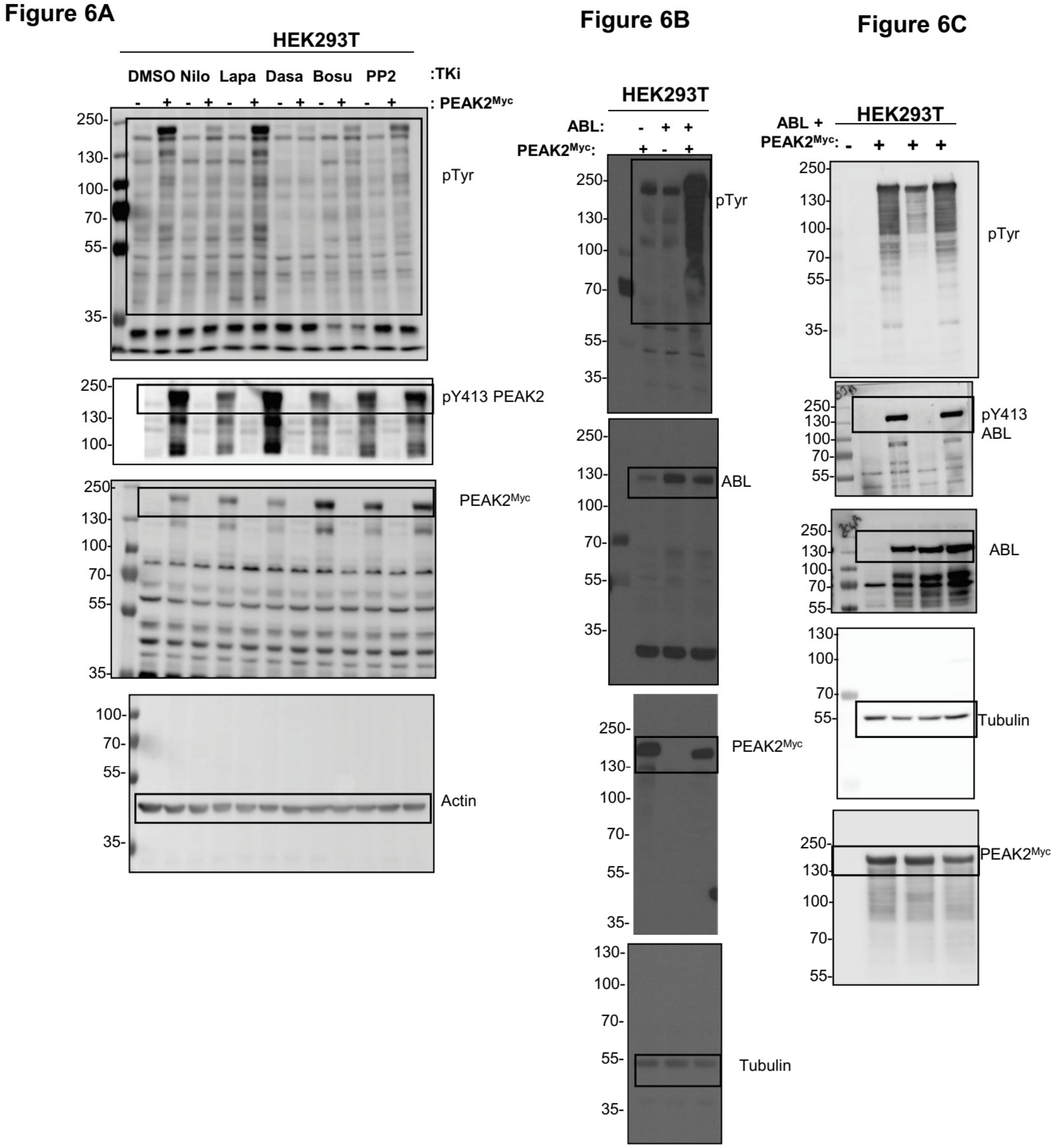
Figure 6A
HEK293T
DMSO Nilo Lapa Dasa Bosu PP2
:TKi
: PEAK2Myc
pTyr
pY413 PEAK2
PEAK2Myc
Actin
Figure 6B
HEK293T
ABL:
PEAK2Myc:
pTyr
ABL
PEAK2Myc
Tubulin
Figure 6C
HEK293T
ABL +
PEAK2Myc:
pTyr
pY413
ABL
ABL
Tubulin
PEAK2Myc

Figure S2A

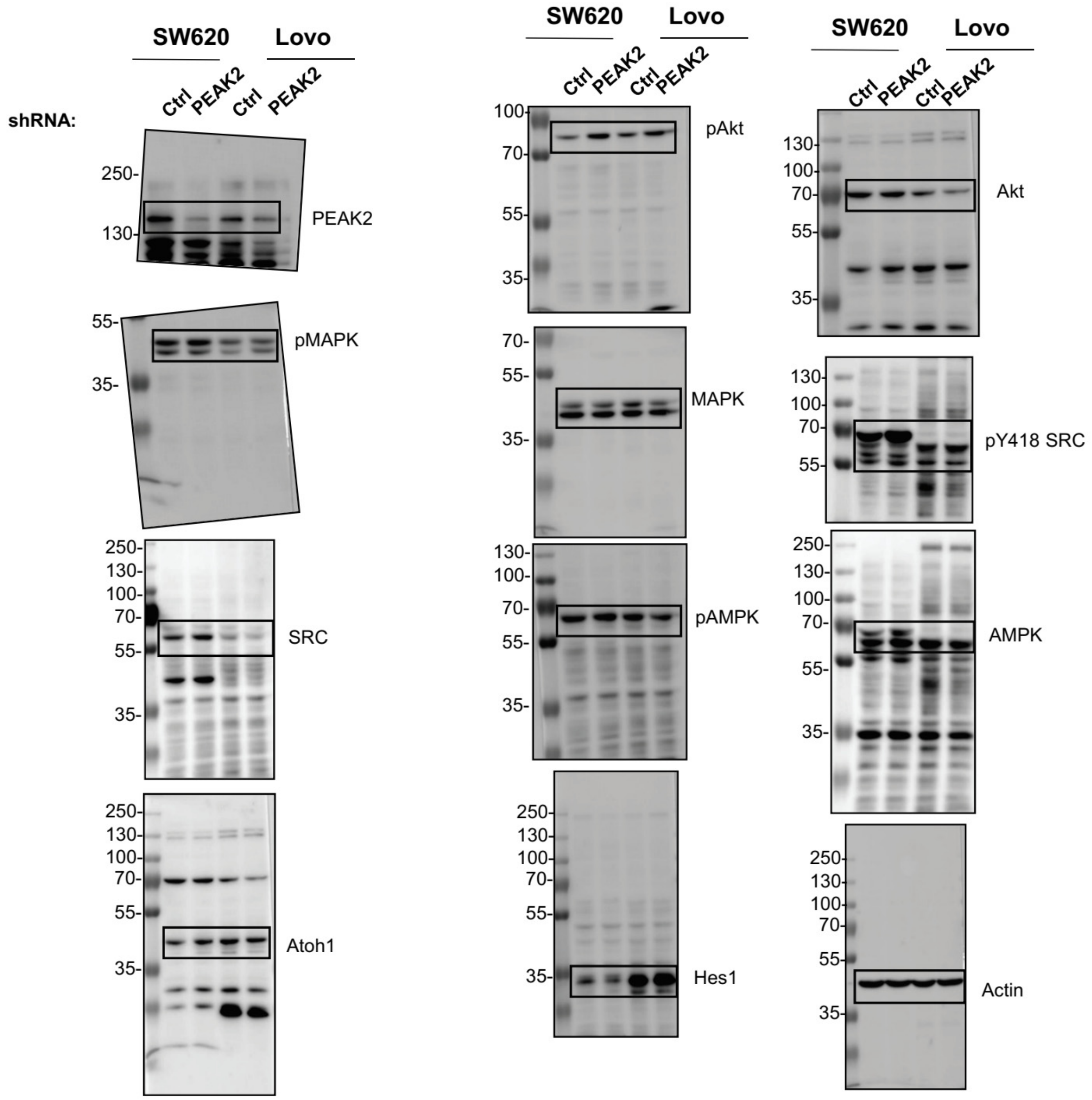

Figure S2B

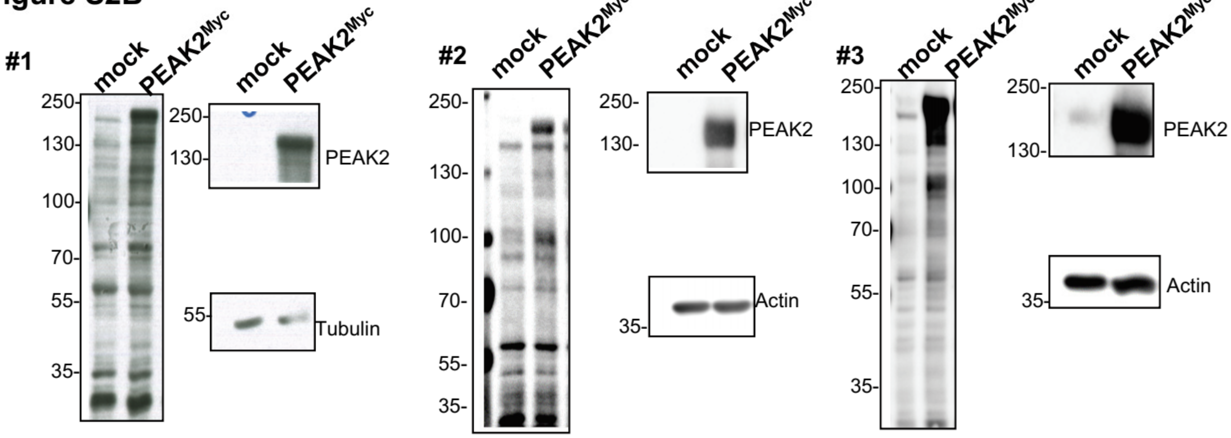

Figure S5

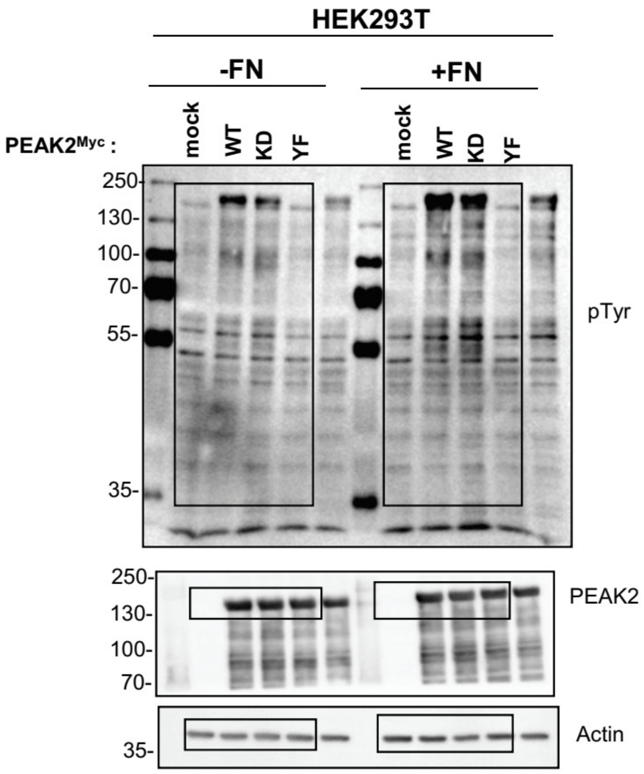

Figure S6. original blots used in this study.
